# Comparative Impact of *Saccharomycodes ludwigii* Inactivation Techniques on the Physicochemical and Sensory Characteristics of Two Spanish White Wines

**DOI:** 10.3390/foods15122165

**Published:** 2026-06-16

**Authors:** Valentina Civa, Ignacio Arias-Pérez, Carolina Castillo-Rio, Purificación Fernández-Zurbano, Maria-Pilar Sáenz-Navajas, Paola Domizio

**Affiliations:** 1 Department of Agriculture, Food, Environment and Forestry (DAGRI), University of Florence, 50144 Firenze, Italy; valentina.civa@unifi.it; 2 Department of Enology, Instituto de Ciencias de la Vid y del Vino (CSIC-GR-UR), Finca La Grajera, 26007 Logroño, La Rioja, Spain; ignacio.arias@icvv.es (I.A.-P.); carolina.castillo@icvv.es (C.C.-R.); puri.fernandez@unirioja.es (P.F.-Z.); 3 Department of Chemistry, Universidad de La Rioja, Instituto de Ciencias de la Vid y el Vino, ICVV (UR-CSIC-GR), Madre de Dios 51, 26006 Logroño, La Rioja, Spain

**Keywords:** yeast derivative, *Saccharomycodes ludwigii*, polysaccharides, colloidal stability, wine protein stability, tartrate stability, sensory evaluation, Flash Profiling

## Abstract

Despite the widespread use of yeast derivatives (YDs) in winemaking, comparative studies on the impact of different inactivation technologies remain limited, especially when applied to non-conventional yeast species. To address this, the present study evaluated the impact of *Saccharomycodes ludwigii* (SL#64) derivatives, a non-conventional species characterized by a high cell wall polysaccharide content, produced via Thermal Inactivation (TI), High-Pressure Processing (HPP), and Partial Lysis combined with HPP (PL + HPP), on the polysaccharide content, colloidal stability, and sensory profiles of Garnacha Blanca and Tempranillo Blanco wines. A commercial *Saccharomyces cerevisiae* derivative was used as a reference yeast derivative. Polysaccharide concentration and colloidal stability were monitored at 2 and 15 days post-addition. All YDs significantly increased polysaccharide levels (3–21%), primarily within the first 48 h. HPP-treated SL#64-YDs proved the most effective in enhancing protein stability, reducing heat-induced turbidity by 21% after 15 days. SDS-PAGE analysis suggested that YD-derived polysaccharides acted as protective colloids, partially maintaining pathogenesis-related (PR) proteins in solution. In contrast, no significant improvement in tartaric stability was observed across treatments. Overall sensory evaluation, including quality, balance, aroma intensity, and Flash Profile characterization, revealed that YD treatments more clearly modified the sensory profile of the aromatically neutral Garnacha Blanca wines, whereas the greater aromatic complexity of the Tempranillo Blanco matrix partially masked these effects, although prolonged contact with TI and HPP reduced aroma intensity and perceived quality. These results suggest that HPP-derived yeast derivatives could be especially suitable for short contact applications, representing a promising non-thermal strategy for tailoring functional YDs to specific wine matrices.

## 1. Introduction

Ensuring colloidal stability remains one of the primary challenges in the production of high-quality white wines. Protein haze and tartrate precipitation are the two most common forms of instability that can lead to significant economic losses and consumer rejection. Historically, bentonite fining has been the standard treatment for protein stabilization. However, its use is often associated with the loss of desirable volatile compounds and wine volume [1]. In recent years, alternative methodologies to bentonite fining have been explored to mitigate white wine protein instability [2,3,4]. Among these, the application of GRAS (Generally Recognized As Safe) proteases, either in free form [5] or immobilized on specific supports such as chitosan beads [6], has been proposed to preserve wine sensory characteristics. Although these enzymatic treatments are promising, the natural resistance of grape PR proteins to proteolysis often requires enzymes to be coupled with high-energy pre-heating steps to unfold the proteins, or the implementation of complex continuous reactors [5,7]. Similarly, cold stabilization for tartrate prevention is energy-intensive and can alter the wine’s sensory equilibrium. To provide winemakers with complementary, easy-to-apply, and sustainable strategies that do not require additional energy inputs, thermal risks, or complex equipment, there is a growing interest in the direct application of yeast derivatives (YDs), such as inactivated yeasts and mannoproteins, as multifunctional and natural tools for colloidal stabilization [8,9]. Mannoproteins, along with other parietal macromolecules naturally released during yeast autolysis, act as protective colloids by inhibiting the aggregation of PR proteins and interfering with the growth of potassium bitartrate crystals [10,11]. However, the effectiveness of YDs is highly dependent on their structural integrity and the molecular weight of their released polysaccharides, which are influenced by the extraction method used. While traditional thermal treatments and enzymatic hydrolysis may cause structural denaturation [12,13,14], High-Pressure Processing (HPP) has emerged as a promising non-thermal alternative [15,16]. HPP can promote the release of polysaccharides while preserving their native conformation, potentially offering enhanced functional properties compared to traditional derivatives. Furthermore, the use of non-*Saccharomyces* species represents an innovative frontier in oenology, as these strains can provide unique polysaccharide profiles that differ from those of traditional *S. cerevisiae* [17,18,19,20]. Following the initial characterization of these novel bio-adjuvants [19], Civa et al. [21] demonstrated that thermally inactivated derivatives from a specific strain of *S. ludwigii* (SL#64) were highly effective in mitigating protein haze in white wine. More recently, this SL#64 strain was revisited to explore its pilot-scale production and evaluate different inactivation methods in wine-like solutions and laboratory-scale fermentations [16]. The selection of this unconventional SL#64 strain is highly novel, as its ability to release large quantities of unique parietal macromolecules makes it a promising bio-adjuvant for wine stabilization, clearly distinguishing it from traditional commercial *S. cerevisiae* derivatives. A critical factor that remains insufficiently explored is the influence of the wine matrix on the functionality of these alternative derivatives. The interaction between yeast polysaccharides and the specific chemical footprint of the grape variety, especially the phenolic composition, can drastically alter the stability outcome. This is particularly relevant for varieties like Tempranillo Blanco, a spontaneous mutation of red Tempranillo, which possesses a distinct and reactive phenolic heritage compared to traditional white varieties like Garnacha Blanca [22].

Based on these premises, the current investigation focuses on the post-fermentation utilization of these specific non-*Saccharomyces* derivatives in real wine matrices. This approach fills a critical gap by shifting the focus from model solutions or fermentation dynamics to the direct interaction between these derivatives and the distinct chemical matrix of finished varietal wines. This study is based on two main hypotheses: first, that non-thermal (HPP) and hybrid Partial Lysis combined with HPP (PL + HPP) inactivation techniques preserve the native functional conformation of SL#64 polysaccharides more effectively than traditional thermal methods, thereby providing enhanced stabilizing properties; and second, that the protective colloid efficiency and sensory impact of these specific derivatives depend strictly on the matrix. Therefore, this study provides a novel comparative assessment of how the specific chemical footprint of different grape varieties modulates the efficiency of differently processed yeast derivatives, comparing the aroma-neutral Garnacha Blanca with the more complex, phenolic-rich Tempranillo Blanco. Specifically, the aim of this work was to elucidate the ‘structure-function’ relationship of these tailored *S. ludwigii* derivatives by evaluating their impact on the protein and tartrate stability, as well as the sensory profile, of these two distinct Spanish white wines.

## 2. Materials and Methods

### 2.1. Yeast Strains and Yeast Derivative Preparation

The non-*Saccharomyces* yeast *S. ludwigii* (SL#64), belonging to the microbial culture collection of DAGRI , was used for yeast derivative (YD) preparations. A commercial inactive *S. cerevisiae* preparation (FN 401, Angel yeast Co., Ltd., Yichang, China) was used as a reference yeast derivative (Table 1). The production of SL#64-YDs followed the procedures described by Civa et al. [16]. Briefly, SL#64 biomass was produced through a three-step cultivation process: an initial pre-culture in YPD medium (24 h, 28 °C, 130 rpm), followed by a batch propagation phase in YED medium using a 5 L bioreactor (24 h, 29 °C, pH 5.0, 500 rpm, 2 L/min aeration), and a final fed-batch propagation phase. This final phase was conducted for up to 24 h at 31 °C and pH 5.4, under 1200 rpm stirring and 1 vvm aeration (dissolved oxygen > 50%), with a continuous supply of glucose solution (210 g/L) and 3% NH_3_. After cooling to 15 °C, the biomass was harvested by centrifugation (4000 rpm, 10 min) and washed twice with deionized water. The resulting yeast cream was then subjected to three distinct inactivation treatments: (i) Thermal Inactivation (TI), achieved via direct spray drying at an inlet temperature of 160 °C and an outlet temperature of 105–110 °C; (ii) High-Pressure Processing (HPP), consisting of two cycles at 5000 bar (5 min each, 4 °C) followed by low-temperature spray drying (inlet: 110 °C; outlet: 70 °C); (iii) PL + HPP treatment. This last inactivation treatment involved a preliminary partial enzymatic lysis aimed at increasing the porosity of the yeast cell wall prior to mechanical disruption. This partial lysis was performed using 5 g/hL of a commercial β-1,3-glucanase preparation, incubated at 40 °C for 2 h to facilitate the release of parietal compounds, such as glucans and mannoproteins. Following enzyme inactivation (90 °C for 20 min), the yeast cream was subjected to High-Pressure Processing.

### 2.2. Wine Treatments and Experimental Design

Two not stabilized white wines (vintage 2023), produced from Spanish varieties Garnacha Blanca and Tempranillo Blanco, were provided by Bodega de la Grajera, belonging to the Government of La Rioja (Logroño, La Rioja, Spain). Basic chemical parameters (Table 2) were analyzed via Fourier Transform Infrared (FTIR) spectrometry (WineScan™ FT 120, FOSS^®^, Hillerød, Denmark).

The experimental design was structured to systematically evaluate the impact of yeast derivative treatments across two distinct wine matrices (aroma-neutral Garnacha Blanca versus aromatic and phenolic-rich Tempranillo Blanco). For each wine, five treatment conditions were compared: an untreated control (CT) and four yeast derivative additions (SC, TI, HPP, PL + HPP) applied at a standard enological dosage of 40 g/hL. To assess the evolution of the treatments, sampling was performed at two specific contact times (2 and 15 days) selected to monitor the immediate release of yeast cell wall macromolecules and their subsequent short-term colloidal interactions within the specific wine matrix. All independent experimental conditions were performed in biological triplicates. The wines were stored in 1.3 L glass bottles sealed with screw caps and kept under strictly controlled temperature conditions (17 ± 1 °C).

### 2.3. Wine Analyses

#### 2.3.1. Quantification of Total Polysaccharides

Total polysaccharides were measured using high-performance liquid chromatography (HPLC), following the protocol outlined in Civa et al. [19]. Briefly, 20 μL of each sample was injected into the HPLC system (Shimadzu CBM-20A, Kyoto, Japan), equipped with a SIL-20A autosampler, a LC-20AD pump, a DGU-20AS degasser, and a RID-20S refractive index (RI) detector. Isocratic separation was conducted on a TSKgel OLIGO-PW (808031) column (30 cm × 7.8 mm i.d.) (Supelco, Bellefonte, PA, USA). The mobile phase consisted of 0.2 M sodium chloride (Sigma-Aldrich, Milan, Italy), flowing at a rate of 0.8 mL/min. Polysaccharides were quantified using an external calibration curve established with mannan (Sigma-Aldrich, Milan, Italy) across a concentration range of 100 to 1000 mg/L. Peak integration and data processing were performed using LabSolutions software version 5.121 (Shimadzu Corporation, Kyoto, Japan ). All analyses were conducted in triplicate.

#### 2.3.2. Evaluation of Protein Stability

Protein stability was evaluated by measuring heat-induced haze according to the protocol described by Pocock and Waters [23]. Briefly, wine samples were filtered through 0.22 μm PTFE membranes and heated at 80 °C for 2 h. Following heat treatment, the samples were cooled at 4 °C for 16 h and then allowed to equilibrate at room temperature for 2 h. Turbidity was subsequently measured using a nephelometer (HI93703 turbidimeter, Hanna Instrument Inc., Woonsocket, RI, USA).

#### 2.3.3. Proteins Profiling by Gel Electrophoresis

Before and after the heat test, the samples were centrifuged, and 10 mL aliquots were mixed with four volumes of cold 95% ethanol containing HCl 0.3 M. The mixture was kept at 4 °C for 16 h to precipitate the polysaccharides and proteins. After centrifugation (9000× *g*, 4 °C, 30 min), the supernatant was discarded, and the pellet was washed twice with four volumes of cold 96% (*v*/*v*) ethanol before being vacuum-dried at room temperature. The dried pellet was then rehydrated with distilled water. A 20 μL aliquot of the sample was treated with 6.65 μL of 4X Laemmli buffer (Bio-Rad Laboratories, Hercules, CA, USA) and 2.75 μL of 1 M dithiothreitol (DTT) (Bio-Rad Laboratories, Hercules, CA, USA), then heated at 95 °C for 5 min. Subsequently, 10 μL of the mixture was loaded onto a 10% SDS–polyacrylamide gel. Proteins were separated using the Mini-PROTEAN II electrophoresis system (Bio-Rad Laboratories, Hercules, CA, USA) according to the method of Laemmli [24]. Molecular weight was determined using Bio-Rad standards (#161-0317, Bio-Rad Laboratories, Hercules, CA, USA).

#### 2.3.4. Evaluation of Tartrate Stability

Tartaric stability was evaluated before and after the treatment using a cold test. Briefly, 15 mL tubes were filled with wine and refrigerated at 4 °C for 15 days. All the analyses were performed in triplicate. Stability was assessed using visual inspection for the presence of crystals at the bottom of the tubes and by calculating the change in tartaric acid concentration, determined via an enzymatic assay (BioSystems Y200, Barcelona, Spain).

#### 2.3.5. Sensory Analysis

Sensory analysis was carried out by a tasting panel comprising 14 established winemakers from the DOCa Rioja region. Panelists had extensive experience in winemaking (between 5 and 39 years). The age range was between 30 and 69 years of age, and there were 7 females and 7 males. Each participant attended two sessions (each of 90 min maximum) on different days, each devoted to evaluating the wines of a specific variety. The testing was conducted at the ICVV in individual purpose-built sensory booths at ambient temperature (22–23 °C) under white fluorescent lighting. Participants gave informed consent, and sensory testing was conducted under the ethics approval of the ethical committee of CSIC number 098/2024.

In each session, participants were presented with 20 mL of each of 11 wine samples in black ISO glasses coded with three-digit numbers and covered with plastic Petri dishes. The samples included five wines from the 2-day contact time treatment, five from the 15-day contact time treatment, and one replicated sample to assess the repeatability of the evaluations. To limit carryover and priming effects, wine samples were monadically presented in a different order specific to each participant according to a Williams Latin square arrangement. Water and pectin (1 g/L) were used in the rinsing protocol. The 11 wines were described by the 14 participants following a five-step procedure: (1) assessment of overall quality, considering aroma, flavor, taste and mouthfeel, using a 10-cm continuous scale anchored from *insufficient* to *excellent*, (2) evaluation of positive aroma intensity on a 10-cm continuous scale anchored from *low* to *high*, with *average* as the midpoint reference; (3) assessment of mouth balance on a 10-cm continuous scale anchored from *low* to *high*; (4) identification of sensory attributes that differentiated the 11 samples; and (5) ranking of the samples according to the attributes generated in step 4, following the Flash Profile methodology.

Positive aroma intensity was defined as the intensity of pleasant orthonasal odors perceived in comparison with a reference wine of average intensity. Mouth balance was associated with the concept of edge, defined as an inadequate level—either excessive or insufficient—of acidity, astringency, or bitterness.

#### 2.3.6. Statistical and Data Analysis

*General Statistical analysis:* Experimental data were subjected to analysis of variance (ANOVA), followed by Tukey’s honest significant difference (HSD) (*p* < 0.05) to determine significant differences between samples. Results are expressed as mean ± standard deviations. To explore relationships among the wine samples, Principal Component Analysis (PCA) and Hierarchical Cluster Analysis (HCA) were performed. All statistical procedures were performed using XLSTAT software (version 2023.3.1, Addinsoft, Paris, France).

*Sensory data analysis*: Ranked data obtained from the Flash Profiling were organized into individual matrices (wines in rows, attributes in columns) for each of the 14 participants. These matrices were merged into a global data matrix and subjected to Generalized Procrustes Analysis (GPA). Only descriptors mentioned by at least 20% of the panel (minimum of three assessors) were included in the final visualization of samples and attributes. A subsequent HCA was performed using the first dimensions derived from the GPA to identify clusters of samples with a similar sensory profile. Finally, a two-way ANOVA was conducted for quality, positive aroma intensity, and mouth balance scores. In this model, participants were treated as a random factor, while the HCA- derived clusters were treated as fixed factors to identify significant sensory differences between groups. These specific sensory analyses were performed using XLSTAT (version 2014.2.02; Addinsoft, NY, USA).

## 3. Results and Discussion

### 3.1. Total Polysaccharides

As shown in Table 3, all wines supplemented with YDs exhibited higher polysaccharide concentrations compared to the control samples. These levels were influenced by both the YD production method and the wine matrix, while the contact time appeared to play a secondary role after the initial 48 h.

Regarding Garnacha Blanca, a significant increase in polysaccharides, ranging from 3% to 21%, was observed in the treated wines compared to the control after just 2 days of contact. Similarly, in Tempranillo Blanco, most treatments led to an increase between 5% and 14%. In both wine varieties, the PL + HPP treatment consistently yielded the highest absolute polysaccharide concentrations, reaching 353.51 mg/L (+22%) in Garnacha Blanca and 306.90 mg/L (+23%) in Tempranillo Blanco by day 15.

Furthermore, while a numerical increase in polysaccharide concentration was observed for all treatments between day 2 and day 15 (particularly for PL + HPP), the release of polysaccharides effectively reaches a steady state within the first 48 h, with the combined treatment PL + HPP emerging as the most effective production method for maximizing polysaccharide enrichment.

The observed increase in polysaccharide concentration in both white wines confirms the efficacy of the *S. ludwigii* (SL#64) strain as an important source of cell wall macromolecules. This corroborates the findings of prior studies conducted in model solutions, which demonstrated its macromolecular potential [16]. Unlike traditional aging on lees, which requires prolonged periods for autolytic cell wall degradation and the subsequent enzymatic solubilization of mannoproteic complexes, the pilot-scale inactivation processes optimized previously [16] facilitate the immediate technological release of these macromolecules into the matrix. Moreover, the consistency between the results of the present study in real wines and those previously reported in model solutions [16] demonstrates the robustness of the production process. This confirms that SL#64-YDs maintain constant and reproducible extraction properties, regardless of the matrix treated, consolidating their potential as rapid and effective tools for the colloidal enrichment of wines.

From a mechanistic standpoint, the effectiveness levels of the inactivation methods recorded in this study closely reflect the structural damage models proposed by Civa et al. [16]. Specifically, the results show that the combination of partial enzymatic lysis and high-pressure mechanical stress (PL + HPP treatment) performed better than individual treatments. The synergy between the two techniques is capable of overcoming cell wall resistance more profoundly than traditional physical disruption. While β-glucanase enzymatic lysis hydrolyzes the glucan network, increasing its overall porosity, the subsequent HPP step physically disrupts the weakened wall structure. In this context, high-pressure processing has been reported to enhance the extraction yield of intracellular and parietal polymers primarily by inducing significant alterations in cell integrity and permeability through mass transfer acceleration [15,25]. This mechanical disruption facilitates the solubilization of not only the outermost, easily releasable mannoproteins, but also the structural polysaccharide fractions more deeply integrated within the inner parietal matrix.

In contrast, the lower extraction efficiency of the thermal inactivation (TI) treatment suggests that the high temperatures typical of spray drying may induce protein denaturation or irreversible structural collapse on the yeast surface, thereby limiting the hydration and solubility of the parietal polymers. This structural modification may also explain the initial decrease in polysaccharide concentration observed in the Tempranillo Blanco matrix. This phenomenon likely arises from a heat-induced ‘barrier effect’ on the yeast surface, which triggers an adsorption process between specific wine matrix components and the thermally altered yeast walls, acting as a non-specific binding site for native wine macromolecules. Alternatively, the specific polysaccharides released by these thermal YDs might interact with other reactive wine components and rapidly precipitate. These findings highlight that, although the release potential is fundamentally dictated by the upstream inactivation method, the final colloidal recovery is strictly modulated by the specific characteristics of the oenological matrix.

### 3.2. Colloidal Interactions and Protein Stability

To evaluate the impact of YDs additions on colloidal stability, heat-induced turbidity (ΔNTU) was analyzed together with electrophoretic profiles (SDS-PAGE) after 2 and 15 days of contact. This integrated approach allowed for a comprehensive characterization of the stabilization mechanisms associated with SL#64-YDs.

**Figure 1:**
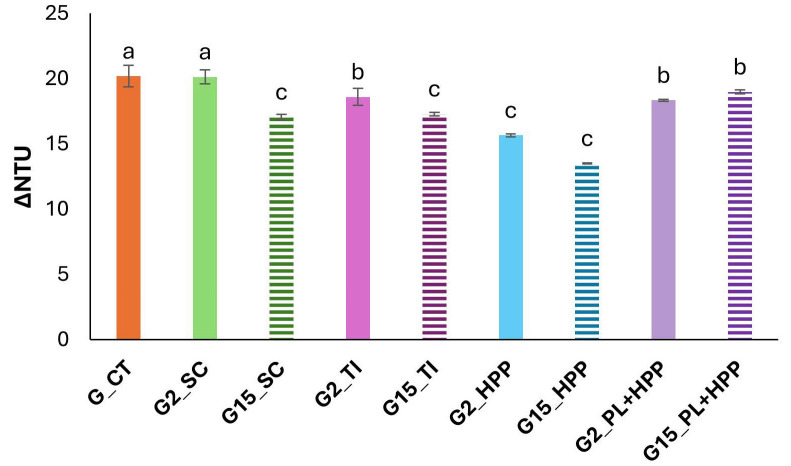
Haziness of Garnacha Blanca 2 days (G2_) and 15 days (G15_) after the addition of YDs; CT: control untreated wine; SC: *S. cerevisiae* derivative; TI: Thermally Inactivated *S. ludwigii* derivative; HPP: High-Pressure Processed *S. ludwigii* derivative; PL + HPP: Partially Lysed + High-Pressure Processed *S. ludwigii* derivative. Data are reported as mean ± standard deviation of three independent replicates. Different letters indicate significantly different values (*p* < 0.05).

For Garnacha Blanca, the control wine exhibited a high susceptibility to protein precipitation with a ΔNTU value of 20.14 (Figure 1). With the exception of the SC treatment at day 2, all SL#64-YD treatments significantly reduced heat-induced haziness. While these reductions were insufficient to guarantee full protein stability (typically defined as ΔNTU < 2), a clear positive trend emerged over time. Specifically, the HPP derivative emerged as the most effective stabilizer, reducing turbidity by 21% after 48 h and reaching a minimum of 13.51 ΔNTU by day 15 (a 33% total reduction). In contrast, the TI and SC treatments showed more modest improvements (14% and 16% respectively at day 15), while the PL + HPP treatment plateaued after an initial 9% decrease.

Interestingly, no direct linear correlation was observed between the total concentration of released polysaccharides and the reduction in heat-induced haze. Although the PL + HPP treatment yielded the highest polysaccharide enrichment, the HPP derivative proved to be more effective in limiting protein aggregation, suggesting that the functional quality and structural integrity of the macromolecules play a more decisive role in protein stabilization than their absolute quantity.

**Figure 2:**
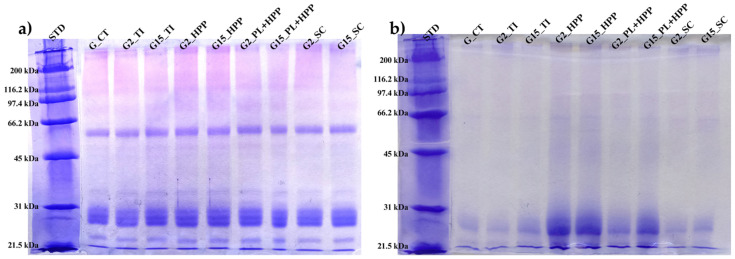
SDS-PAGE electrophoresis of Garnacha Blanca wine proteins after 2 (G2) and 15 days (G15) of contact with YDs (**a**) before heat test; (**b**) after heat test. CT: control untreated wine; SC: *S. cerevisiae* derivative; TI: Thermally Inactivated *S. ludwigii* derivative; HPP: High-Pressure Processed *S. ludwigii* derivative; PL + HPP: Partially Lysed + High-Pressure Processed *S. ludwigii* derivative.

The electrophoretic analysis of Garnacha Blanca samples, before the heat test (Figure 2a), identified distinct bands between 21.5 kDa and 31 kDa, likely corresponding to grape PR proteins, such as thaumatin-like proteins and chitinases, which are widely recognized as the primary drivers of protein haze [26]. Post-heat treatment (Figure 2b), a marked reduction in these bands was observed in the control, likely due to thermal unfolding and subsequent insolubilization; however, lanes corresponding to HPP and PL + HPP treatments maintained significantly high-intensity bands in this region. This directly supports the hypothesis that the specific polysaccharides released by these non-thermal treatments act as protective colloids, maintaining PR proteins in solution and preventing their nucleation into visible aggregates.

**Figure 3:**
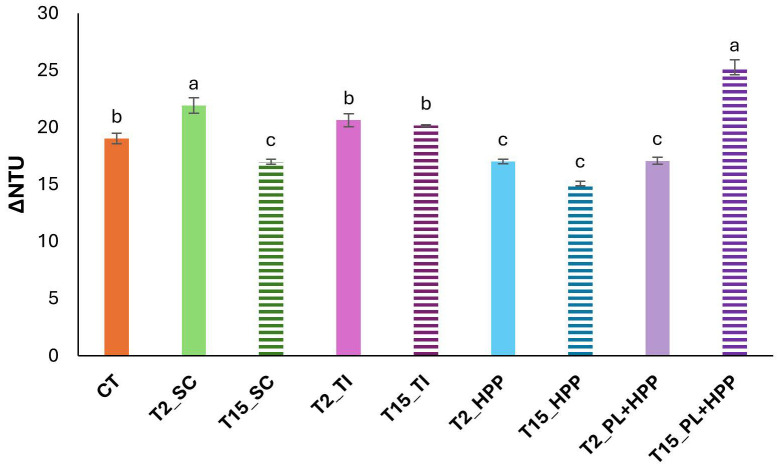
Haziness of Tempranillo Blanco 2 days (2_) and 15 days (15_) after the addition of YDs; CT: control untreated wine; SC: *S. cerevisiae* derivative; TI: Thermally Inactivated *S. ludwigii* derivative; HPP: High-Pressure Processed *S. ludwigii* derivative; PL + HPP: Partially Lysed + High-Pressure Processed *S. ludwigii* derivative. Data are reported as mean ± standard deviation of three independent replicates. Different letters indicate significantly different values (*p* < 0.05).

**Figure 4:**
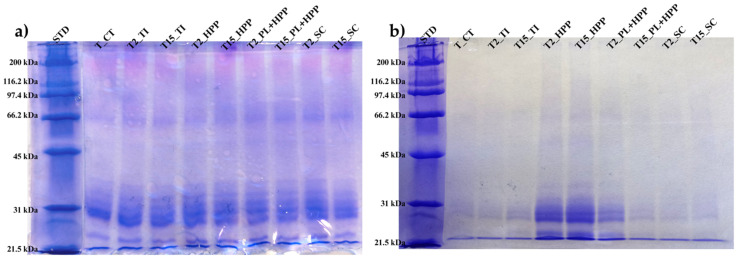
SDS-PAGE electrophoresis of Tempranillo Blanco wine proteins after 2 (T2) and 15 days (T15) of contact with YDs (**a**) before heat stability test; (**b**) after heat test. CT: control untreated wine; SC: *S. cerevisiae* derivative; TI: Thermally Inactivated *S. ludwigii* derivative; HPP: High-Pressure Processed *S. ludwigii* derivative; PL + HPP: Partially Lysed + High-Pressure Processed *S. ludwigii* derivative.

Also in Tempranillo Blanco, the relationship between polysaccharide release and haze reduction proved to be non-linear and highly matrix-dependent (Figure 3). Unlike Garnacha, the SC treatment initially increased turbidity (+15% at day 2) before showing a decrease by day 15. The PL + HPP treatment exhibited an initial 10% reduction in haze followed by a 33% increase after 15 days, suggesting a potential loss of colloidal equilibrium or the formation of unstable ternary complexes over time. Consistent with the findings in Garnacha, the HPP treatment remained the most effective stabilization tool, reducing turbidity by 21% after 15 days. Electrophoretic profiles for Tempranillo (Figure 4a,b) showed similar trends to those observed in Garnacha. In both cases, the PR-protein bands remained visible after thermal stress in the HPP and PL + HPP samples, confirming the solubilizing effect of these SL#64-YDs macromolecules.

The divergence in protein haze reduction between Garnacha Blanca and Tempranillo Blanco underscores the strong influence of the wine matrix on YD effectiveness. Although higher polysaccharide concentrations are generally associated with improved stability in enology [4], our data confirm that macromolecular conformation is more critical than absolute mass. According to the structural profiles established by Civa et al. [16], while PL + HPP maximizes the release of the largest polymeric fractions (F1: 200–805 kDa), the HPP treatment targets a more balanced distribution centered in the intermediate F2 fraction (113–200 kDa). The superior and longer-lasting performance of HPP in both varieties suggests that this intermediate molecular range provides the optimal spatial configuration for effective steric hindrance against protein-protein aggregation. Furthermore, the significant difference in performance between HPP and TI, despite their overlapping molecular weight profiles [16], indicates that high-pressure processing preserves the native, highly-hydrated hydrocolloidal structure of the polysaccharides, which is instead likely compromised by thermal denaturation during spray drying.

The divergent behavior observed in Tempranillo Blanco further highlights the role of the varietal matrix. This cultivar is characterized by an uncommonly high phenolic potential for a white variety. As reported by Gascueña et al. [22], Tempranillo Blanco originates from a spontaneous genetic mutation of red Tempranillo, inheriting a distinct and highly reactive phenolic heritage compared to traditional white cultivars like Garnacha Blanca. This inherent phenolic richness likely facilitates the formation of unstable ternary complexes between grape proteins, yeast-derived polysaccharides, and varietal polyphenols, mirroring the macromolecular interactions observed by Quijada-Morín et al. [27] in Tempranillo Tinto.

In this context, the excessive extraction of large, highly-branched polymers (F1 fraction) typical of the PL + HPP treatment may initially coat the proteins, but over 15 days, it promotes bridging flocculation. In this scenario, the oversized polysaccharides bind multiple protein and polyphenol units simultaneously, acting as a molecular bridge that grows until it exceeds its solubility limit, leading to the observed increase in turbidity. By contrast, HPP treatment provides a balanced and compact molecular profile that resists bridging even in polyphenol-rich environments, ensuring a more persistent protective colloid effect.

These findings therefore establish high-pressure processing (HPP) as the most effective technology for protein stabilization, providing a consistent balance between molecular weight, structural integrity and long-term colloidal stability.

### 3.3. Tartrate Stability

The tartrate stability of Garnacha Blanca and Tempranillo Blanco wines was evaluated following cold stabilization by measuring the concentration of tartaric acid (Δ Tartaric acid, g/L) (Table 4). The data reveal that the addition of yeast derivatives did not produce a uniform stabilizing effect nor one directly proportional to the concentration of polysaccharides present.

In the case of Garnacha Blanca, the control wine exhibited a tartaric acid loss of 0.11 g/L. Most SL#64-YDs treatments did not significantly improve the initial situation. On the contrary, the sample treated with the commercial derivative (SC) after 15 days showed a significantly higher precipitation (0.27 g/L), suggesting potential negative interference or higher instability under those specific conditions. Although the PL + HPP treatment at day 2 showed the numerically lowest precipitation value (0.07 g/L), this difference was not statistically significant compared to the control, confirming the absence of a clear technological benefit for this variety. Similarly, no treatment in Tempranillo Blanco significantly outperformed the control (0.11 g/L). Although the 15_HPP sample reached a lower value (0.04 g/L), this improvement was not statistically significant.

Although specific mannoproteins are widely recognized for their potential to inhibit tartrate crystal growth by adsorbing onto active crystal nuclei faces [11], this study found no direct correlation between the total concentration of polysaccharides released and the prevention of tartrate precipitation. This indicates that the mere presence of yeast macromolecules is insufficient to guarantee tartaric stability. This lack of efficacy under our experimental conditions may be attributed to the specific composition of the Garnacha and Tempranillo matrices. Native components, such as specific low-molecular-weight polyphenols or an unfavorable potassium/calcium mineral balance, may have competitively hindered the functional active sites of the mannoproteins, preventing them from effectively blocking crystal growth steps.

### 3.4. Sensory Evaluation

**Figure 5:**
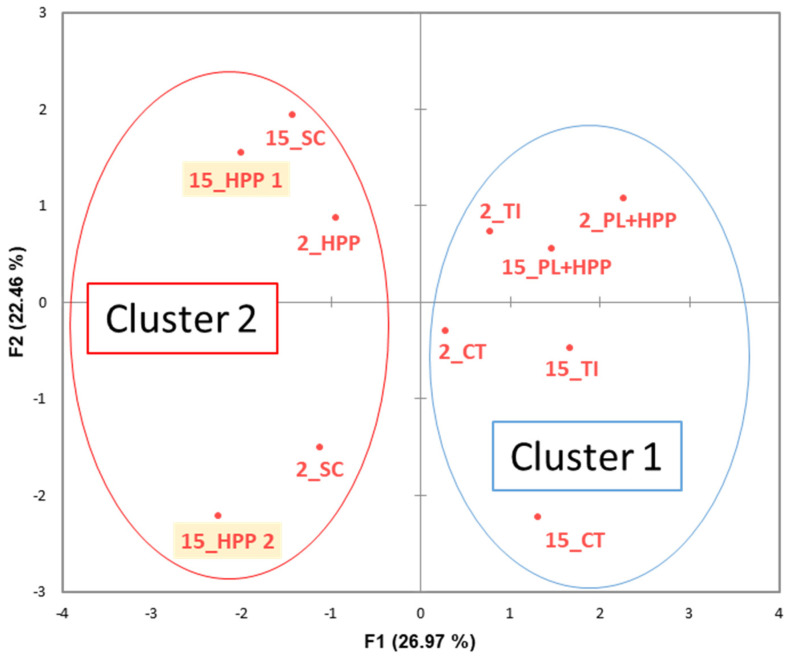
Projections of Garnacha Blanca samples on the plane formed by the first two dimensions obtained in the Generalized Procrustes Analysis (GPA) carried out on the sensory data obtained in the Flash profiling. CT: control untreated wine; SC: *S. cerevisiae* derivative; TI: Thermally Inactivated *S. ludwigii* derivative; HPP: High-Pressure Processed *S. ludwigii* derivative; PL + HPP: Partially Lysed + High-Pressure Processed *S. ludwigii* derivative.

The Flash Profile analysis was performed to evaluate the sensory profiles resulting from the different YD treatments after 2 and 15 days of contact. Figure 5 shows the GPA projections of Garnacha Blanca samples on the first two principal components, which explained 26.97% and 22.46% of the total variance, respectively. The samples were separated into two main clusters. Replicated samples (15_HPP1 and 15_HPP2, highlighted in yellow in the figure) were projected within the same cluster, confirming the good repeatability of the sensory evaluations.

Cluster 1 (blue) comprised the untreated control wines (CT) together with samples treated with the SL#64-YDs after thermal inactivation (TI) and after partial lysis plus high-pressure processing (PL + HPP). These wines were associated with positive sensory attributes, including acidity, volume, and fruity and floral notes. In contrast, Cluster 2 (red), which included wines treated with the *S. cerevisiae* derivative (SC) and the SL#64 YD after high-pressure processing (HPP), was mainly characterized by bitterness and significantly lower overall quality scores (F = 1.994; *p* < 0.05) than samples of Cluster 1.

**Figure 6:**
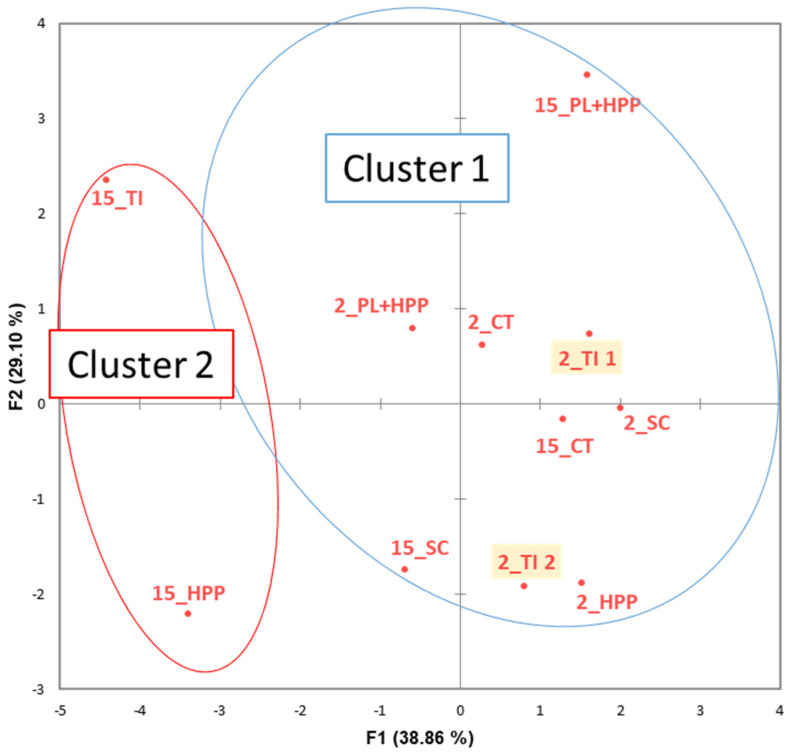
Projections of Tempranillo Blanco samples on the plane formed by the first two dimensions obtained in the Generalized Procrustes Analysis (GPA) carried out on the sensory data obtained in the Flash Profiling. CT: control untreated wine; SC: *S. cerevisiae* derivative; TI: Thermally Inactivated *S. ludwigii* derivative; HPP: High-Pressure Processed *S. ludwigii* derivative; PL + HPP: Partially Lysed + High-Pressure Processed *S. ludwigii* derivative.

Figure 6 shows the GPA projections of Tempranillo Blanco samples. The first two principal components explained 38.86% and 29.10% of the total variance, respectively. Similar to Garnacha Blanca, hierarchical cluster analysis (HCA) separated the samples into two distinct groups. The replicated samples (2_TI1 and 2_TI2, highlighted in yellow in the figure) were projected within the same cluster, confirming the good repeatability of the sensory analysis.

Cluster 1 (blue) included the control wines (CT), samples treated with PL + HPP and SC derivatives, as well as wines treated for 2 days with TI and HPP derivatives. In contrast, Cluster 2 (red) grouped wines treated for 15 days with TI and HPP derivatives. These results indicate that, unlike Garnacha Blanca, contact time influenced the sensory perception of Tempranillo Blanco wines, particularly for the TI and HPP treatments.

No specific sensory descriptors clearly discriminated between the two clusters, likely due to the intrinsically intense aromatic profile of Tempranillo Blanco, characterized by strong fruity aromas that may have masked more subtle treatment-related differences. Nevertheless, panelists consistently perceived a decrease in positive aroma intensity in the 15-day treatments grouped in Cluster 2, which was accompanied by a significant reduction in perceived overall quality. Accordingly, wines in Cluster 1 received significantly higher scores for both overall quality (F = 4.105; *p* < 0.05) and positive aroma intensity (F = 3.585; *p* < 0.05) than wines in Cluster 2.

Although previous studies have reported positive effects of commercial mannoproteins on wine sensory quality [28] and their ability to modulate aroma volatility via hydrophobic interactions [29], no direct linear relationship was observed between the total concentration of polysaccharides released and the sensory clusters identified here. As highlighted by Pozo-Bayón et al. [30], the impact of YDs on wine aroma is a dynamic equilibrium dependent on contact time, as yeast cell wall matrices can actively adsorb volatile compounds, particularly hydrophobic esters and long-chain volatile fatty acids.

In the present work, this adsorption capacity appeared to depend heavily on both the specific wine matrix and the micro-structural state of the applied treatment. In Garnacha Blanca, wines treated for 2 and 15 days were generally grouped together, indicating that the structural nature of the YD treatment had a greater impact on sensory perception than contact time. In contrast, Tempranillo Blanco wines treated with TI and HPP derivatives showed clear chronological separation, where the 15-day treatments experienced a significant drop in positive aroma intensity and lower perceived quality.

The absence of clearly discriminating sensory descriptors in Tempranillo Blanco may be related to the intrinsically intense aromatic profile of this variety, characterized by dominant fruity notes that could mask more subtle treatment-related differences. Nevertheless, panelists consistently perceived a reduction in aroma intensity in the 15-day treatments, suggesting that prolonged contact with these specific SL#64 formulations may promote a “scalping” effect (i.e., sorption and retention) on the volatile fraction, wherein the open structure of the non-thermalized walls or the porous nature of the thermalized ones selectively fix varietal volatile compounds. The intense, fruit-forward aromatic profile of Tempranillo Blanco, while initially masking subtle changes, rendered this variety highly vulnerable to aroma loss over extended contact times.

Overall, these findings highlight the critical importance of considering varietal aroma profile and wine composition when evaluating the sensory impact of yeast derivative treatments. Further studies focused on the molecular interactions between YDs and specific wine matrices could help optimize their industrial use for targeted colloidal and sensory modulation.

## 4. Conclusions

This study provides the first comprehensive evaluation of *S. ludwigii* derivatives (SL#64-YDs) obtained through innovative extraction technologies, offering a novel biotechnological approach to improve wine stability. The findings demonstrate that supplementing with these derivatives represents a promising tool, although its effectiveness depends on the extraction method and the specific wine matrix. Among the treatments, High-Pressure Processing (HPP) proved to be the most reliable technology for long-lasting protein stabilization, thanks to the release of a balanced polysaccharide profile (F2 fraction, 113–200 kDa) acting as a protective colloid. By contrast, the partial lysis combined with HPP (PL + HPP) induced delayed colloidal destabilization in Tempranillo Blanco, demonstrating that excessive extraction of high-molecular-weight polymers (F1 fraction) can cause flocculation with varietal polyphenols. Regarding tartaric stability, the macromolecular enrichment failed to inhibit crystal growth, likely due to a lack of structural features like phosphorylation.

Sensory analysis revealed that the impact of SL#64-YDs is driven by the qualitative composition of the macromolecules rather than their quantity. Furthermore, the primary aroma profile of the grape variety modulated this perception: the derivatives were easily discriminated in the neutral Garnacha Blanca, whereas the intense fruity aroma of Tempranillo Blanco exerted a masking effect. Additionally, prolonged contact time (15 days) in Tempranillo Blanco reduced positive aroma intensity, potentially due to volatile compound adsorption.

A main limitation of this study is that it focused only on two specific wine matrices over a limited contact time, which restricts the immediate generalization of these findings to other varieties. Moreover, the exact molecular mechanisms driving the interaction between specific polysaccharide fractions, volatile compounds, and phenolics remain to be fully clarified.

Overall, this work highlights for the first time the potential and challenges of using non-*Saccharomyces* derivatives, showing that their application must be carefully calibrated according to the grape variety and matrix. Further studies are required to overcome these limitations and ensure that improvements in protein stability do not promote delayed colloidal instability.

## Figures and Tables

**Table 1:** Yeast strains utilizd for YDs production.

Strain	Species	Origin	YD Code
FN401	*Saccharomyces cerevisiae*	Angel Yeast ^a^	SC
#64	*Saccharomycodes ludwigii*	DAGRI ^b^	TI
#64	*Saccharomycodes ludwigii*	DAGRI ^b^	HPP
#64	*Saccharomycodes ludwigii*	DAGRI ^b^	PL + HPP

^a^ Angel yeast Co., Ltd. (Yichang, China). ^b^ Department of Agriculture, Food, Environment and Forestry (DAGRI), University of Florence, Florence, Italy. SC: *S. cerevisiae* derivative; TI: Thermally Inactivated *S. ludwigii* derivative; HPP: High-Pressure Processed *S. ludwigii* derivative; PL + HPP: Partially Lysed + High-Pressure Processed *S. ludwigii* derivative.

**Table 2:** Basic chemical parameters of Garnacha Blanca and Tempranillo Blanco.

Wine	Alcohol % (*v*/*v*)	Titratable Acidity (Tartaric Acid g/L)	Volatile Acidity (Acetic Acid g/L)	pH
Garnacha Blanca	13.03	5.50	0.26	3.30
Tempranillo Blanco	12.78	6.40	0.30	3.46

**Table 3:** Total polysaccharide concentration (mg/L) in Garnacha Blanca and Tempranillo Blanco 2 days (2_) and 15 days (15_) after the addition of YDs.

Sample	Garnacha Blanca	Tempranillo Blanco
CT	289.52 ^d^ ± 10.15	249.05 ^cd^ ± 6.72
2_SC	303.36 ^cd^ ± 5.96	260.99 ^bc^ ± 12.25
15_SC	316.68 ^c^ ± 8.94	267.03 ^bc^ ± 9.78
2_TI	298.35 ^cd^ ± 3.45	233.55 ^d^ ± 13.64
15_TI	306.73 ^cd^ ± 4.88	252.06 ^cd^ ± 6.50
2_HPP	322.80 ^bc^ ± 6.82	264.46 ^bc^ ± 5.61
15_HPP	325.43 ^abc^ ± 13.91	273.14 ^bc^ ± 11.02
2_PL + HPP	351.03 ^ab^ ± 4.40	283.96 ^ab^ ± 6.87
15_PL + HPP	353.51 ^a^ ± 9.74	306.90 ^a^ ± 1.67

CT: control untreated wine; SC: *S. cerevisiae* derivative; TI: Thermally Inactivated *S. ludwigii* derivative; HPP: High-Pressure Processed *S. ludwigii* derivative; PL + HPP: Partially Lysed + High-Pressure Processed *S. ludwigii* derivative. Data are reported as mean ± standard deviation of three independent replicates. Different letters within a column indicate significantly different values (*p* < 0.05).

**Table 4:** Δ Tartaric acid (g/L) of the samples after 2 (2_) and 15 days (15_) of the treatments.

Sample	Garnacha Blanca	Tempranillo Blanco
CT	0.11 ^b^ ± 0.02	0.11 ^ab^ ± 0.02
2_SC	0.18 ^ab^ ± 0.03	0.10 ^ab^ ± 0.02
15_SC	0.27 ^a^ ± 0.09	0.07 ^ab^ ± 0.03
2_TI	0.12 ^b^ ± 0.02	0.08 ^ab^ ± 0.02
15_TI	0.18 ^ab^ ± 0.06	0.14 ^a^ ± 0.02
2_HPP	0.15 ^ab^ ± 0.02	0.09 ^ab^ ± 0.04
15_HPP	0.11 ^ab^ ± 0.04	0.04 ^b^ ± 0.02
2_PL + HPP	0.07 ^b^ ± 0.05	0.12 ^a^ ± 0.04
15_PL + HPP	0.11 ^b^ ± 0.04	0.09 ^ab^ ± 0.03

CT: control untreated wine; SC: *S. cerevisiae* derivative; TI: Thermally Inactivated *S. ludwigii* derivative; HPP: High-Pressure Processed *S. ludwigii* derivative; PL + HPP: Partially Lysed + High-Pressure Processed *S. ludwigii* derivative. Data are reported as mean ± standard deviation of three independent replicates. Different letters indicate significantly different values (*p* < 0.05).

## Data Availability

The original contributions presented in the study are included in the article, further inquiries can be directed to the corresponding authors.

## Abbreviations

The following abbreviations are used in this manuscript:

G_CT	Garnacha Blanca wine without treatment
G2_PL + HPP	Garnacha Blanca wine treated for 2 days with *S. ludwigii* derivative inactivated with a partial enzymatic treatment and pressure processing
G2_HPP	Garnacha Blanca wine treated for 2 days with *S. ludwigii* derivative inactivated with pressure processing
G2_SC	Garnacha Blanca wine treated for 2 days with a commercial *S. cerevisiae* derivative
G2_TI	Garnacha Blanca wine treated for 2 days with *S. ludwigii* derivative inactivated with a thermal treatment
G15_PL + HPP	Garnacha Blanca wine treated for 15 days with *S. ludwigii* derivative inactivated with a partial lysis treatment and pressure processing
G15_HPP	Garnacha Blanca wine treated for 15 days with *S. ludwigii* derivative inactivated with a pressure processing
G15_SC	Garnacha Blanca wine treated for 15 days with a commercial *S. cerevisiae* derivative
G15_TI	Garnacha Blanca wine treated for 15 days with *S. ludwigii* derivative inactivated with a thermal treatment
SL#64-YD	*S. ludwigii* strain #64 derivative
T2_PL + HPP	Tempranillo Blanco wine treated for 2 days with *S. ludwigii* derivative inactivated with a partial lysis treatment and pressure processing
T2_HPP	Tempranillo Blanco wine treated for 2 days with *S. ludwigii* derivative inactivated with a pressure processing
T2_SC	Tempranillo Blanco wine treated for 2 days with a commercial *S. cerevisiae* derivative
T2_TI	Tempranillo Blanco wine treated for 2 days with *S. ludwigii* derivative inactivated with a thermal treatment
T15_PL + HPP	Tempranillo Blanco wine treated for 15 days with *S. ludwigii* derivative inactivated with a partial enzymatic treatment and pressure processing
T15_HPP	Tempranillo Blanco wine treated for 15 days with *S. ludwigii* derivative inactivated with a pressure processing
T15_SC	Tempranillo Blanco wine treated for 15 days with a commercial *S. cerevisiae* derivative
T15_TI	Tempranillo Blanco wine treated for 15 days with *S. ludwigii* derivative inactivated with a thermal treatment
